# Germline mutations in Chinese ovarian cancer with or without breast cancer

**DOI:** 10.1002/mgg3.1940

**Published:** 2022-05-24

**Authors:** Ava Kwong, Cecilia Yuen Sze Ho, Vivian Yvonne Shin, Chun Hang Au, Wing Pan Luk, Ling Hiu Fung, Tsun‐Leung Chan, Karen Kar Loen Chan, Hextan Yuen Sheung Ngan, Edmond Shiu Kwan Ma

**Affiliations:** ^1^ Department of Surgery The University of Hong Kong Pofulam Hong Kong SAR; ^2^ Department of Surgery University of Hong Kong‐Shenzhen Hospital Shenzhen China; ^3^ Department of Surgery Hong Kong Sanatorium & Hospital Happy Valley Hong Kong SAR; ^4^ Hong Kong Hereditary Breast Cancer Family Registry Shau Kei Wan Hong Kong SAR; ^5^ Department of Molecular Pathology Hong Kong Sanatorium & Hospital Happy Valley Hong Kong SAR; ^6^ Department of Medical Physics and Research Hong Kong Sanatorium & Hospital Happy Valley Hong Kong SAR; ^7^ Department of Obstetrics and Gynaecology The University of Hong Kong Pofulam Hong Kong SAR

**Keywords:** Chinese, germline mutation, hereditary breast and ovarian cancer

## Abstract

**Background:**

Ovarian and breast cancers are known to have significant genetic components. Considering the differences in the mutation spectrum across ethnicity, it is important to identify hereditary breast and ovarian cancer (HBOC) genes mutation in Chinese for clinical management.

**Methods:**

Two cohorts of 451 patients with ovarian cancer only (OV) and 93 patients with both breast and ovarian (BROV) cancers were initially screened for *BRCA1, BRCA2, TP53*, and *PTEN*. 109 OV and 43 BROV patients with extensive clinical risk and were being tested negative, were then further characterized by 30‐gene panel analysis.

**Results:**

Pathogenic *BRCA1/2* variants were identified in 45 OV patients and 33 BROV patients, giving a prevalence of 10% and 35.5%, respectively. After the extended screening, mutations in other HBOC genes were identified in an additional 12.8% (14/109) of the OV cohort and 14% (6/43) in the BROV cohort. The most commonly mutated genes in the OV cohort were *MSH2* (4.6%) while in the BROV cohort were *MSH2* (4.7%) and *PALB2* (4.7%). With this extended multigene testing strategy, pathogenic mutations were detected in 12.8% of OV patients (*BRCA*s: 10%; additional genes: 12.8%) and 40.9% (*BRCA*s: 35.5%; additional genes: 14%) of BROV patients.

**Conclusion:**

Extended characterization of the contributions of HBOC genes to OV and BROV patients has significant impacts on further management in patients and their families, expanding the screening net for more asymptomatic individuals.

## Graphical abstract

Extended characterization of the contributions of HBOC genes to patients with ovarian cancer and patients with both breast and ovarian cancers have significant impacts on further management in patients and their families, expanding the screening net for more asymptomatic individuals.

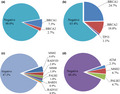

## 1 BACKGROUND

Hereditary breast and ovarian cancer (HBOC) syndrome is an inherited cancer predisposition syndrome causing breast and ovarian cancer at higher risk than the general population. Individuals with HBOC also have an increased risk of other types of cancers, including pancreatic cancer, prostate cancer, and melanoma (Petrucelli et al., 1998). *BRCA1/2* plays key roles in homologous recombination (HR) which govern the repair of double‐strand DNA breaks (Roy et al., 2011) and mutations in these two genes are the most common cause of the syndrome (Stanislaw et al., 2016). Breast cancer is the most common malignancy in individuals with a germline *BRCA1/2* pathogenic variant, resulting in a lifetime risk ranging from 46% to 87% (Chen et al., 2006; Ford et al., 1994; Sankaran et al., 2006). *BRCA* germline pathogenic variants also confer an excessive risk for ovarian cancers (including fallopian tube and primary peritoneal cancers) ranging from 16.5% to 63% (Chen et al., 2006; Easton et al., 1995). Mutations in *BRCA1/2* genes were detected in approximately 12%–25% of patients with high‐grade serous ovarian carcinoma (HGSC) (Chao et al., 2016; Sakamoto et al., 2016; Walsh et al., 2011) while the prevalence of *BRCA1/2* mutation was (3.7%–4.7%) in women with breast cancer, aged between 40 and 59 years (Buys et al., 2017). The *BRCA1/2* mutation rate in 18 Chinese cohorts with metachronous BROV malignancies was as high as 38.9% (Chao et al., 2020). There were well‐established counseling strategies and management guidelines for early intervention or prevention through increased surveillance, such as clinical breast examination every 6–12 months and addition of annual breast MRI, mammography at age 25, and consideration of risk‐reducing interventions including chemoprevention and prophylactic mastectomy or salpingo‐oophorectomy (Petrucelli et al., 1998; Smith, 2012).

Multiple cancer predisposition genes, other than *BRCA1/2* genes, have been identified during the last decade to the deliberate risk of breast or ovarian cancer, such as *TP53*, *PTEN*, *CDH1*, *ATM*, *CHEK2*, *PALB2*, and *RAD50* (Apostolou & Fostira, 2013; Bradbury & Olopade, 2007; Kwong et al., 2020; Ripperger et al., 2009). Due to the rapid advancement and reduced cost in high‐throughput sequencing, multigene panel testing becomes a standard risk assessment tool in clinical practice (Bradbury et al., 2015; Domchek et al., 2013; Fecteau et al., 2014; Lincoln et al., 2015; Minion et al., 2015; Rainville & Rana, 2014; Tung et al., 2015). Together an with increased number of clinical trials and recommendations from National Comprehensive Cancer Network (NCCN) guidelines on other cancer predisposition genes, high‐risk individuals, particularly those with strong family history and an early onset of cancer are more prevalent. Hence, strategic selection for genetic screening in Chinese ovarian cancer patients is required to identify patients with high cancer risk.

In this study, we compared the results of mutation screening we adopted earlier on the 4 most common HBOC genes and combined data with a more recent strategy using 30 gene‐panel between women with ovarian cancers (OV) only and women with both breast and ovarian cancers (BROV). We observed a difference in the spectra of mutated genes in these 2 cohorts which is important in clinical management and planning of screening strategies for patients and their family members.

## 2 METHODS

### 2.1 Ethical compliance

The study was performed following the Declaration of Helsinki. Written informed consent was obtained from all participants recruited in this study. This study was approved by the Institutional Review Board of the University of Hong Kong/Hospital Authority West Cluster and respective authorities of other contributing hospitals in Hong Kong.

### 2.2 Patients

A cohort of 451 patients with OV cancer and 93 patients with both BROV cancer regardless of age and family history were recruited by the Hong Kong Hereditary and High‐risk Breast Cancer Family Registry from 2012 to 2018. Clinicopathologic characteristics of patient cohorts were shown in Table S1. Known *BRCA1/2* mutations (positive control) and normal local individuals (negative control) were included for validation of the next generation sequencing (NGS) and to evaluate the performance characteristics of NGS.

### 2.3 DNA extraction

Genomic DNA for NGS or conventional Sanger sequencing were extracted from peripheral blood samples with QIAamp DNA Blood Mini Kit (Qiagen, Hilden, Germany) or QIAsymphony DNA Mini Kit (Qiagen) on QIAsymphony SP instrument (Qiagen), according to the manufacturer's protocol. Extracted DNA was quantified using Qubit dsDNA BR Assay Kit and Qubit 2.0 Fluorometer (Life Technologies, USA).

### 2.4 Four‐gene and 30‐gene sequencing panel

**FIGURE 1:**
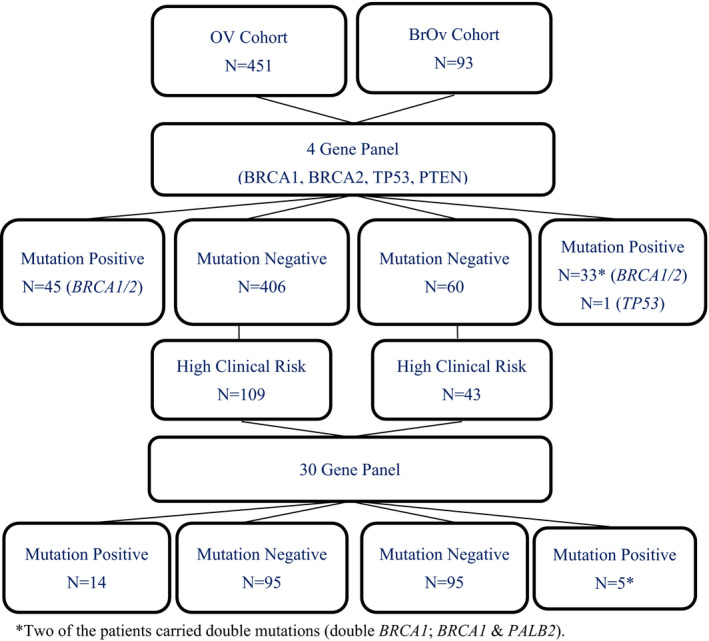
Schematic diagram of study samples

Extracted DNAs (OV cohort: 451; BROV: 93) were first sequenced with a 4‐gene panel including *BRCA1*, *BRCA2*, *TP53*, and *PTEN* using a previously published in‐house protocol (Kwong et al., 2016). Those negative cases with extensive clinical risk (OV cohort: 109; BROV cohort: 43) were selected for further analysis with a 30‐gene test panel (Color Genomics Laboratory, Burlingame, CA) (Figure 1). This panel includes genes that are associated with an elevated risk of a number of hereditary cancers in breast, ovarian, uterine/endometrial, colorectal, melanoma, pancreatic, prostate, and stomach (Neben et al., 2019). The list of genes includes *APC* (OMIM: 611731), *ATM* (OMIM: 607585), *BAP1* (OMIM: 603089), *BARD1* (OMIM: 601593), *BMPR1A* (OMIM: 601299), *BRCA1* (OMIM: 113705), *BRCA2* (OMIM: 600185), *BRIP1* (OMIM: 605882), *CDH1* (OMIM: 192090), *CDK4* (OMIM: 123829), *CDKN2A (p14ARF* and *p16INK4a)* (OMIM: 600160), *CHEK2* (OMIM: 604373), *EPCAM* (OMIM: 185535), *GREM1* (OMIM: 603054), *MITF* (OMIM: 156845), *MLH1* (OMIM: 120436), *MSH2* (OMIM: 609309), *MSH6* (OMIM: 600678), *MUTYH* (OMIM: 604933), *NBN* (OMIM: 602667), *PALB2* (OMIM: 610355), *PMS2* (OMIM: 600259), *POLD1* (OMIM: 174761), *POLE* (OMIM: 174762), *PTEN* (OMIM: 601728), *RAD51C* (OMIM: 602774), *RAD51D* (OMIM: 602954), *SMAD4* (OMIM: 600993), *STK11* (OMIM: 602216), and *TP53* (OMIM: 191170). The majority of these genes were assessed for variants within all coding exons (±20 bp flanking each exon) and noncanonical splice regions. In *PMS2*, exons 12–15 were not analyzed. In several genes, only specific positions known to impact cancer risk were analyzed: *CDK4* ‐ only chr12:g.58145429–58,145,431 (codon 24), *MITF*—only chr3:g.70014091 (including c.952G > A), *POLD1*—only chr19:g.50909713 (including c.1433G > A), *POLE*—only chr12:g.133250250 (including c.1270C > G), *EPCAM*—only large deletions and duplications including the 3′ end of the gene, and *GREM1*—only duplications in the upstream regulatory region (reference to GRCh37, hg19).

### 2.5 Massive parallel DNA sequencing

Sequencing libraries from the above‐mentioned panels were synthesized and purified as previously described (Kwong et al., 2016; Neben et al., 2019). Pooled libraries were sequenced on MiSeq or NextSeq 500/550 instrument (Illumina, San Diego, CA). In the case of splicing variant analysis at the transcript level, RNA samples were reverse transcribed to cDNA. All detected pathogenic variants were further validated by conventional Sanger bi‐directional DNA sequencing. The sequencing data were co‐analyzed by our in‐house developed bioinformatics pipeline.

### 2.6 Variant interpretation and annotation

Variants calling bioinformatics were performed as previously described (Kwong et al., 2016; Neben et al., 2019). Paired sequencing reads were mapped to human reference genome sequence GRCh37/hg19. Variants with a minor allele frequency of at least 1% reported by The 1000 Genomes Projects (1000 Genomes Project Consortium et al., 2015) were excluded from manual variant curation. Variants were described according to the recommendations of the Human Genome Variation Society (HGVS). Variant descriptions were further cross‐checked with Mutalyzer Name Checker (http://mutalyzer.nl).

### 2.7 Statistical analysis

Clinicopathological variables from pathogenic/likely pathogenic mutation carriers and non‐carriers were tabulated in contingency tables. Computation was performed using R (version 3.6.0). Statistical tests suitable for categorical data were then considered. Some variables had expected values of less than 5, and most variables did not have a natural ordering. Fisher's exact test was adopted. Conditional Inference Tree (in R package party kit) was also applied to obtain significant factors among others in predicting mutation. Disease‐free survival (DFS) and overall survival (OS) analysis was done on OV patients with surgical information and follow‐up data. The cox‐proportional hazard model was conducted on the differences in survival across groups with adjustment of other variables. Significance level was set at 5% (*p* value < .05).

## 3 RESULTS

### 3.1 Patients' characteristics of the cohort of ovarian cancers only (OV) and the cohort of breast and ovarian cancers (BROV)

In a cohort of 451 probands with OV only, the median age at diagnosis was 47 years (range 9–78). Of these, 407 (90.2%) were cancers found in the ovary, 27 (6%) were primary peritoneal cancer, 9 (2%) were developed in the fallopian tube, also 8 (1.8%) found synchronous primary gynecological cancers. The majority of the ovarian cancers were epithelial cancer (421, 96.3%) in which many of them were serious tumors (161, 36.9%) and endometrioid tumors (145, 33.3%). Slightly more than half of these tumors were high‐grade cancers (274, 63.3%). A positive family history of breast cancer (first‐ or second‐degree relatives) was seen among 92 (20.4%) of these patients. There were 27 (6%) and 140 (31%) of them having family histories of ovarian cancers or other *BRCA*‐related cancers in the first‐ or second‐degree relatives. Detailed clinicopathological characteristics of the OV cohort were shown in Table S1a.

In the second cohort of 93 patients with BROV cancer, the median age at diagnosis was 45 years (range 18–73), which was similar to the OV cohort. Of those gynecological cancers, 79 (84.9%) were cancers of the ovary, 6 (6.5%) were primary peritoneal cancer, 6 (6.5%) were originated from the uterus and 2 (2.2%) were developed in the fallopian tubes. The majority of cancers were epithelial ovarian cancer (84, 96.6%) of which about half of them were serious tumors (42, 48.8%), followed by endometrioid cancer (24, 27.9%). There was a higher proportion of high‐grade ovarian cancers (60, 75%). With respect to the profile of their breast cancers, probands were mostly diagnosed with ductal breast carcinoma (60, 70.6%). Of which 9 of them had medullary, lobular, or mucinous carcinoma (10.6%), and 16 (18.8%) had ductal carcinoma in situ (DCIS). There was a high percentage of breast cancers that were hormonal positive (40, 43%), HER2 positive (7, 7.5%), and 15 (16.1%) were triple‐negative breast cancers (TNBC). The majority of breast cancers were diagnosed at early stages (0, I or II) (76, 86.4%) and only 31 of the breast cancers (41.3%) were high‐grade invasive breast cancers. A positive family history of breast cancer (first‐ or second‐degree relatives) was seen among 36 (38.7%) of the patients. 14 (15.1%) and 29 (31.2%) of them had a family history of ovarian cancers or other *BRCA*‐related cancers in their first‐ or second‐degree relatives. Detailed clinicopathological characteristics of the BROV cohort were shown in Table S1b.

### 3.2 Mutations in the heredity breast and ovarian cancers (HBOC) genes in the cohorts

**FIGURE 2:**
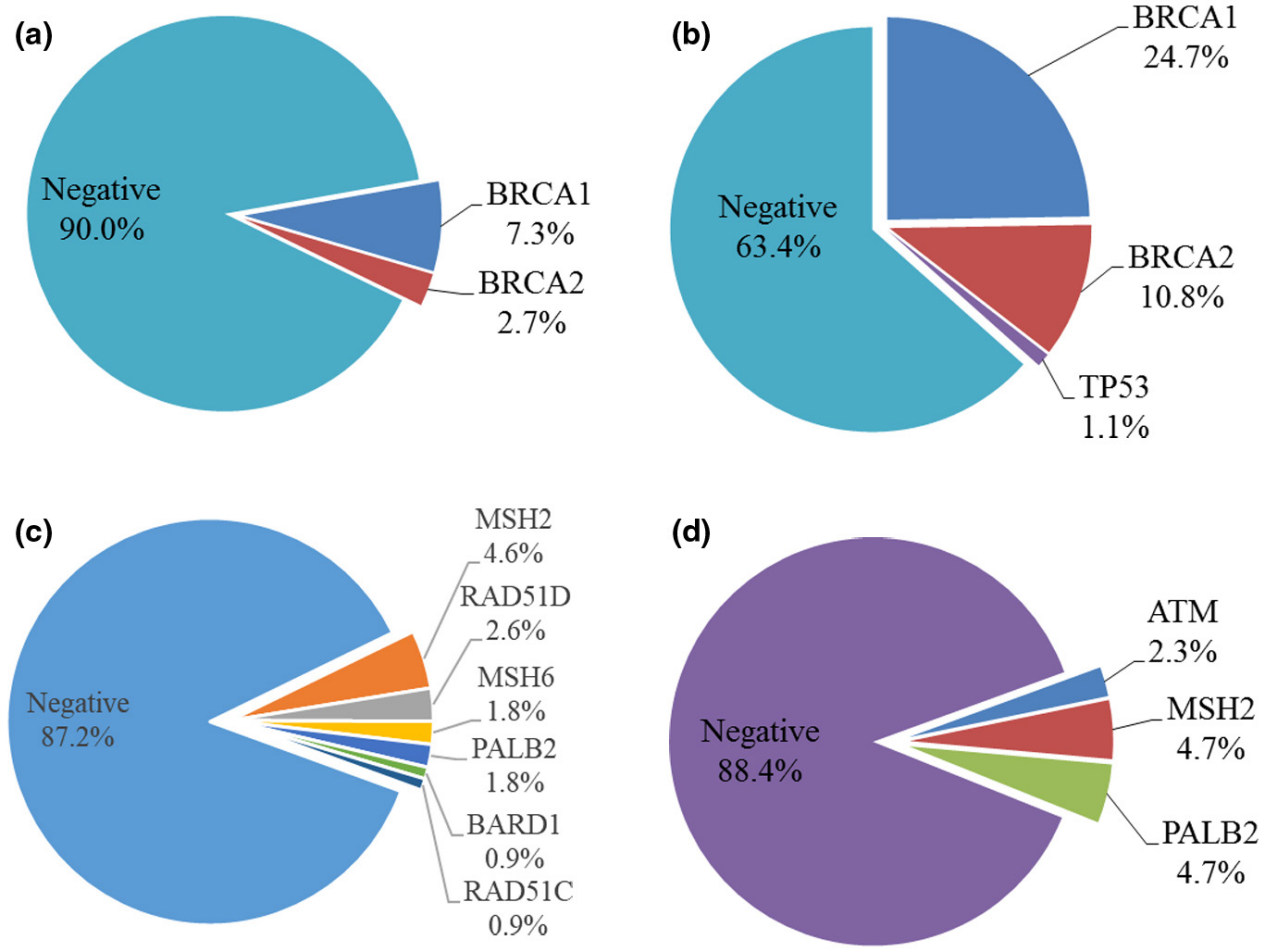
Mutation distributions in ovarian cancers (OV) cohort and breast and ovarian cancers (BROV) cohort. (a) Mutation distributions of the 4‐genes panel in ovarian cancers (OV) cohort (*N* = 451); (b) Mutation distributions of the 4‐genes panel in breast and ovarian cancers (BROV) cohort (*N* = 93). (c) Mutation distributions of the 30‐genes panel in ovarian cancers (OV) cohort (*N* = 109); (d) Mutation distributions of the 30‐genes panel in breast and ovarian cancers (BROV) cohort (*N* = 43)

In the OV cohort, the total numbers of pathogenic mutations were found in 59 of the patients. Among these patients, most of them were *BRCA* mutations 45/451 (10%) and 14/109 (12.8%) harbored mutations other than *BRCA1/2* genes (Figure 2a). Patients with pathogenic mutations in genes other than *BRCA*s included *MSH2* (*n* = 5), *RAD51D* (*n* = 3), *PALB2* (*n* = 2), *MSH6* (*n* = 2), *BRAD1* (*n* = 1), and *RAD51C* (*n* = 1). (Figure 2c).

On the contrary, the total numbers of pathogenic mutations were found in 38 probands in the BROV cohort. 33/93 (35.5%) probands had *BRCA*s mutations and 1/93 (1.1%) was *TP53* mutation carrier using 4‐gene panel (Figure 2b). Two of these probands carried double mutations (double *BRCA1*; *BRCA1* & *PALB2*). The frequency of *BRCA* mutations was much higher than those in the OV cohort (35.5% vs 10%). There were 5/43 (11.6%) probands identified with pathogenic mutations by a 30‐gene panel including *MSH2* (*n* = 2), *PALB2* (*n* = 2), and *ATM* (*n* = 1) as shown in Figure 2d. The 4‐gene panel was able to identify 36.6% of patients with pathogenic mutations, while the extended 30‐gene panel detected the remaining 5.4% among the whole BROV cohort.

There was no significant difference at the age of diagnosis for the mutation carriers and non‐carriers in the OV cohort. The majority of the tumors were diagnosed at a later stage (*p* value < .001) and with higher grade (*p* value < .001) compared with non‐carriers. The site of cancers and histological subtypes of serous and endometrioid cancer showed a significant difference between mutation carriers and non‐carriers (*p* value = .0456, < .001 and .001, respectively). Mutation carriers had a stronger family histories of breast cancers (35.6% vs 18.1%), ovarian cancers (16.9% vs 4.3%), and other *BRCA*‐related cancers (among first‐ and second‐degree relatives) (45.8% vs 28.8%) than non‐carriers (*p* value = .003, .001 and .011, respectively). Detailed characteristics of the pathogenic mutation carriers were shown in Table S1a. There were significant differences in cancer sites, histology, stage, grade, and family history of breast or ovarian cancer in *BRCA* carriers and non‐carriers. However, other mutation gene carriers (beyond *BRCA1/2*) only showed significant differences in family history in *BCRA‐*related cancers when compared with non‐carriers (Table S2a).

Among the BROV cohort, the median age at diagnosis for the mutation carriers and non‐carriers were 43 years and 48 years (*p* value = .030), respectively. The majority of the ovarian tumors were diagnosed at a later stage and with higher grade, compared with non‐carriers (*p* value = .021 and 0.044). Many of the gynecological tumors in these mutation carriers were serous in histology (28, 75.7%, *p* value < .001). Most of the breast tumors were TNBC (12, 31.6%, *p* value = .001). Moreover, positive family histories of breast cancer, ovarian cancer, and other *BRCA*‐related cancers (among first and second‐degree relatives) were seen in 21 (55.3%), 10 (26.3%) and 17 (44.7%) mutation carriers, respectively; compared with 15 (27.3%), 4 (7.3%), and 12 (21.8%) in non‐carriers (*p* value = .009, .017 and .024, respectively). Detailed characteristics of the BROV probands that carried pathogenic mutations were shown in Table S1b. *BRCA* carriers have a younger diagnosis age (≤50), cancer specifically occurs in the ovary with high‐grade and high‐stage serous ovarian cancer. Significant stronger family histories of breast or ovarian cancer were also seen. No significant difference was identified in breast histology. However, beyond *BRCA1/2* carriers only showed significant differences in cancer sites when compared with non‐carriers (Table S2b).

The median age of the first diagnosis of cancers in the OV cohort and the BROV cohort was 50 years and 43 years (*p* value = .020), respectively. The majority of *BRCA1/2* carriers in the BROV cohort had a significant earlier age of diagnosis (≤50 of age) than in the OV cohort (*p* value = .005). *BRCA1/2* mutation carriers in the BROV probands had a stronger family history of breast cancer than the OV probands (*p* value = .040) (Table 1).

**TABLE 1:** Mutation frequency of *BRCA*s and genes other than *BRCAs*

	Ovarian cancer probands (*N* = 451)		Breast and ovarian cancer probands (*N* = 93)		*p* value^a^			
	*BRCA* mutation Carriers (*n* = 45)	Beyond *BRCA* mutation carriers (*n* = 14)	*BRCA* mutation carriers (*n* = 33^a^)	Beyond *BRCA* mutation carriers (*n* = 6^a^)	Ov BRCA vs Ov beyond BRCA	BrOv BRCA vs BrOv beyond BRCA	Ov BRCA vs BrOv BRCA	Ov beyond BRCA vs BrOv beyond BRCA
Age, median (range, yr)	50 (17–74)	42.5 (30–59)	43 (30–73)	38 (22–46)	.0155	.4492	.0199	1
Diagnosis age < 50	22 (48.9%)	12 (85.7%)	27 (81.8%)	6 (100.0%)	.0279	.5669	.0045	1
Ovarian cancer stage								
Stage I	2 (4.5%)	4 (28.6%)	7 (21.2%)	4 (66.7%)	.0449	.1560	.1230	.3182
Stage II	4 (9.1%)	2 (14.3%)	2 (6.1%)	0 (0%)				
Stage III	26 (59.1%)	4 (28.6%)	16 (48.5%)	2 (33.3%)				
Stage IV	12 (27.3%)	4 (28.6%)	6 (18.2%)	0 (0%)				
Not stated	1	0	2	0				
Ovarian cancer site								
Ovarian	35 (77.8%)	13 (92.9%)	27 (81.8%)	3 (50%)	.2390	.0025	.8948	.0103
Fallopian tube	3 (6.7%)	0 (0%)	1 (3%)	0 (0%)				
Peritoneal	6 (13.3%)	0 (0%)	5 (15.2%)	0 (0%)				
Uterus	0 (0%)	0 (0%)	0 (0%)	3 (50%)				
Mixed	1 (2.2%)	1 (7.1%)	0 (0%)	0 (0%)				
Ovarian cancer histology								
Epithelial	45 (100%)	12 (100%)	32 (100%)	6 (100%)	NA	NA	NA	NA
Germ cell	0 (0%)	0 (0%)	0 (0%)	0 (0%)				
Stromal	0 (0%)	0 (0%)	0 (0%)	0 (0%)				
Mixed	0 (0%)	0 (0%)	0 (0%)	0 (0%)				
Not stated	0	2	1	0				
Ovarian cancer histology subtype								
Serous	39 (86.7%)	4 (33.3%)	27 (84.4%)	2 (33.3%)	.0006	.0094	.7507	1
Non serous	6 (13.3%)	8 (66.7%)	5 (15.6%)	4 (66.7%)				
Not stated	0	2	1	0				
Ovarian cancer histology subtype								
Endometrioid	3 (6.7%)	5 (41.7%)	4 (12.5%)	4 (66.7%)	.0074	.0053	.4338	.2941
Non Endometrioid	42 (93.3%)	7 (58.3%)	28 (87.5%)	2 (33.3%)				
Not stated	0	2	1	0				
Ovarian cancer grade								
1	0 (0%)	1 (8.3%)	1 (3.3%)	1 (16.7%)	.0277	.0064	.64	.4134
2	1 (2.3%)	2 (16.7%)	0 (0%)	2 (33.3%)				
3	43 (97.7%)	9 (75%)	29 (96.7%)	3 (50%)				
Not stated	1	2	3	0				
Family history of cancer								
Breast	17 (37.8%)	4 (28.6%)	21 (63.6%)	1 (16.7%)	.7506	.0142	.0395	.5304
Ovarian	9 (20.0%)	1 (7.1%)	10 (30.3%)	1 (16.7%)	.4249	.3067	.4264	1
BRCA related	16 (35.6%)	11 (78.6%)	14 (42.4%)	3 (50.0%)	.0063	.6443	.4872	.5696
Breast cancer stage								
Stage 0	NA	NA	1 (3.4%)	2 (33.3%)	NA	.0962	NA	NA
Stage I	NA	NA	14 (48.3%)	2 (33.3%)				
Stage II	NA	NA	9 (31%)	2 (33.3%)				
Stage III	NA	NA	5 (17.2%)	0 (0%)				
Stage IV	NA	NA	0 (0%)	0 (0%)				
Not stated	NA	NA	4	0				
Breast cancer histology								
Ductal	NA	NA	26 (89.7%)	4 (66.7%)	NA	.0717	NA	NA
Non‐ductal	NA	NA	2 (6.9%)	0 (0%)				
In situ	NA	NA	1 (3.4%)	2 (33.3%)				
Not stated	NA	NA	4	0				
Breast cancer grade								
Low	NA	NA	11 (44%)	2 (40%)	NA	1	NA	NA
High	NA	NA	14 (56%)	3 (60%)				
Not stated	NA	NA	8	1				
Breast cancer molecular subtype								
HER2+	NA	NA	2 (6.1%)	0 (0%)	NA	1	NA	NA
ER/PR+	NA	NA	8 (24.2%)	3 (50.0%)	NA	.1438	NA	NA
TNBC	NA	NA	12 (36.4%)	0 (0%)	NA	.1521	NA	NA

^a^ One of the patient carried both BRCA and PALB2 mutation, clinical data was counted in both BRCA and Beyond BRCA group but was excluded in Fisher's exact test.

When comparing the mutations carriers of *BRCA1/2* and those with mutations in genes other than *BRCA1/2* in the OV cohort, the median age of the first diagnosis of cancer were 50 years and 42.5 years, respectively, showing a statistically significant difference (*p* value = .016). The majority of beyond *BRCA1/2* carriers have an early diagnosis less than or equal to 50 years of age, showing a significant difference (*p* value = .028) compared with *BRCA1/2* carriers. *BRCA1/2* mutations were associated with a higher stage and higher grade of disease when compared with other cancer‐associated gene mutations (*p* value = .045 & .028). *BRCA1/2* mutations were more commonly identified in serous epithelial cancers and a non‐endometrioid tumor, while patients with a mutation in genes other than *BRCA* mutations were seen in neither serous nor endometrioid tumor (*p* value = .001, *p* value = .007, respectively) (Table 1). Beyond *BRCA1/2*, carriers also have a stronger family history of *BRCA‐*related cancers (*p* value = .006).

Similar patterns were also seen in the BROV cohort, *BRCA1/2* mutations were both commonly identified in serous epithelial cancers and non‐endometrioid tumors while a majority of the patients with mutations in genes besides *BRCA1/2* mutations were seen in non‐serous but in endometrioid cancers (*p* value = .009, *p* value = .005 respectively). *BRCA1/2* mutations were associated with a higher grade of disease when compared with other cancer‐associated gene mutations (*p* value = .006). In cases with mutations in *BRCA*, most of their gynecological cancers were developed inside the ovary, and patients with mutations in genes other than *BRCA1/2* mutation in the BROV probands, their gynecological cancers were developed in either ovary or uterus (*p* value = .003) shown in Table 1. No significance different in breast histology in the BROV cohort was observed.

Comparing the mutation carriers in genes besides *BRCA1/2* in the OV cohort and carriers in the BROV cohort, there was no significant difference in the age of diagnosis, histology, and cancer stage and grade (data not shown). The majority of the tumors in the OV cohort developed in the ovary while only half of the tumors in the BROV cohort were from the ovary (*p* value = .010) (Table 1). *MSH2* and *PALB2* mutation carriers were seen in both OV and BROV cohorts. The positive rate of *MSH2* was similar in both cohorts, while the positive rate for *PALB2* was higher in the BROV cohort. Mutation carrier of *ATM* was only found in the BROV cohort but not in the OV cohort (Figure 2c,d).

**FIGURE 3:**
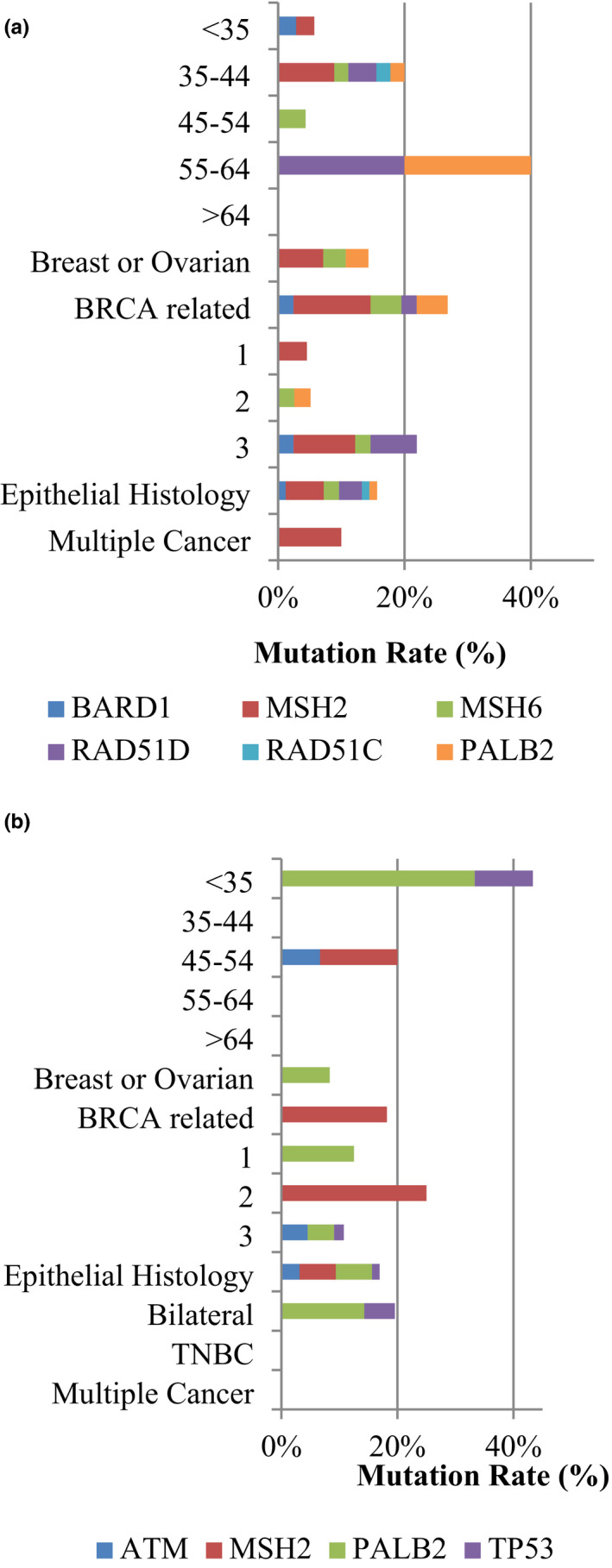
Clinicopathological data of ovarian cancers (OV) and breast and ovarian cancers (BROV) cohort's mutation carriers. Data expressed the percentage of mutation in different genes in different age of diagnosis (Dx age), in different family history background, and personal medical background. Family history of BRCA‐related cancers includes: family history of cancers in colon, stomach, and prostate, and family history of melanoma, cholangiocarcinoma, and pancreatic cancers. Multiple cancers: patient with any other cancers and gynecologic cancers; TNBC: triple‐negative breast cancer; Bilateral: bilateral breast cancer. (a) Clinicopathological data in 14 ovarian cancer (OV) cohort's mutation carriers. *BARD1*; *MSH2; MSH6; RAD51D; RAD51C; PALB2*. (b) Clinicopathological data in 6 breast and ovarian cancers (BROV) cohort's mutation identified by further 30 genes panel. *ATM*; *MSH2*; *PALB2*; *TP53*

Interestingly, the *BRCA1/2*‐positive rate was similar among different age groups in the OV cohort, which was different from the BROV cohort, as well as the *BRCA*‐positive breast cancer patients in our previous study (Kwong et al., 2009). The majority of these two groups of cancer patients were diagnosed at the age between 20 and 44 or later (Table S3a). Families with both breast and ovarian cancer history were more likely to carry *BRCA1/2* mutation than those with either a family history of breast cancers or family history of ovarian cancer history (*p* value < .001). Moreover, families with ovarian cancer history showed a significantly higher mutation rate in genes other than *BRCA1/2* (*p* value = .001) (Table S3b). Correlation of patients with mutations in genes besides *BRCA1/2* with demographic data of the OV cohort and the BROV cohort were illustrated in Figure 3a,b.

**FIGURE 4:**
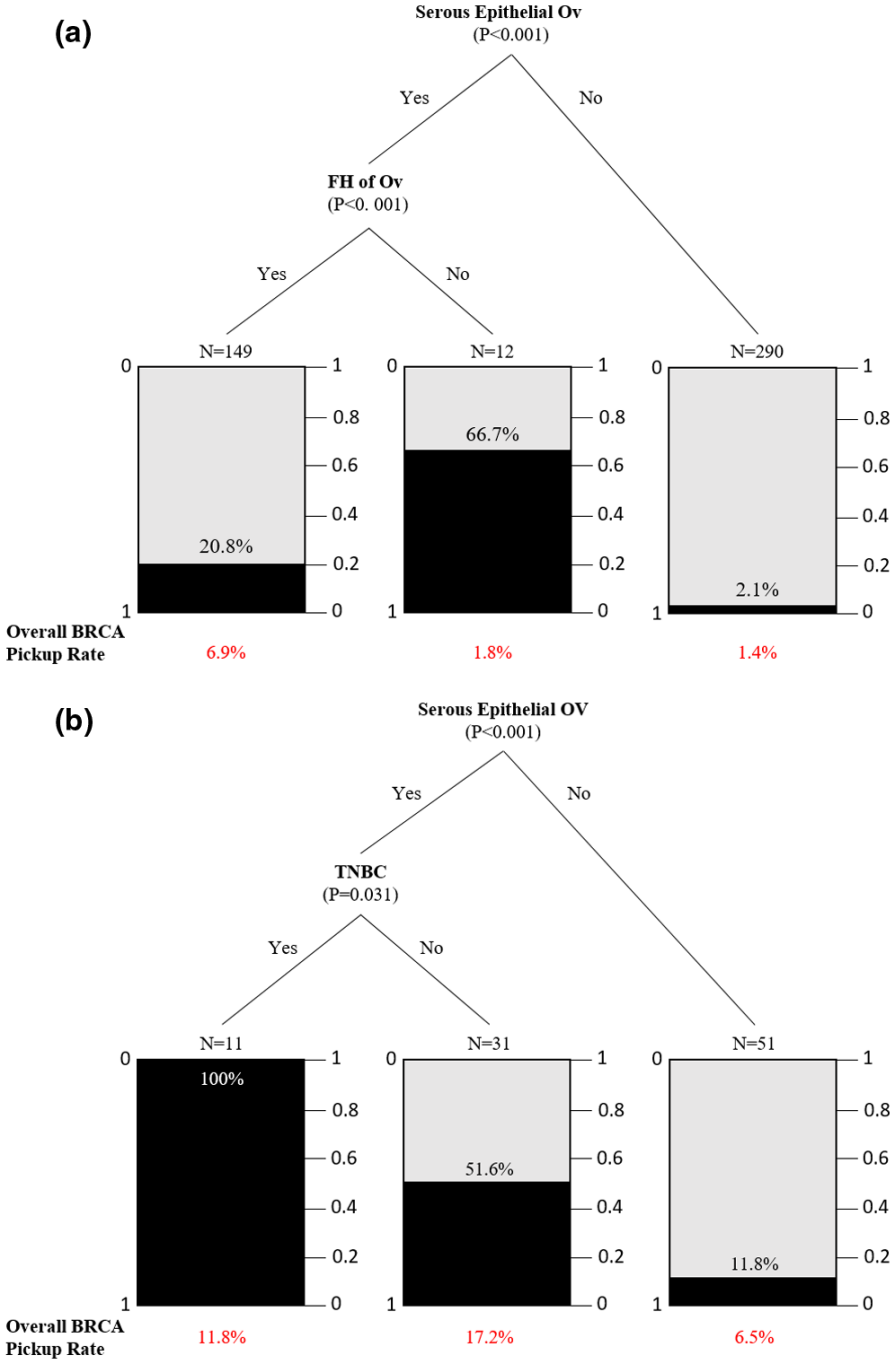
Regression tree for prediction of probability to find *BRCA*s pathogenic mutation carriers. Ov: Ovarian cancers; FH: Family history. (a) Prediction of probability to find *BRCA*s pathogenic mutation carriers with ovarian cancers (OV). (b) Prediction of probability to find *BRCA*s pathogenic mutation carriers with breast and ovarian cancers (BROV)

Classification and regression tree modeling were performed on clinicopathological variables for the OV cohort. The variables included first diagnosis age, personal cancers other than breast and ovarian, site of cancer, histology, grade, stage, family history of breast/ovarian cancer in first‐ and second‐degree relatives. It has already been reported that most of the *BRCA* mutations were found in probands with serous epithelial ovarian cancer (Manchana et al., 2019) and this has also been seen in our cases. Apart from the serous epithelial ovarian cancer, patients with a family history of ovarian cancer were more likely to carry *BRCA1/2* mutations regardless of their family histories. Histological subtypes appeared not pivotal and were shown to be independent of age in this cohort (Figure 4a). In the BROV cohort, patients with serous epithelial ovarian cancer with triple‐negative breast cancer were more likely to carry a *BRCA1/2* mutation (Figure 4b). This provided strong evidence that histological diagnosis needs to be taken into serious consideration in ovarian cancer patients.

Among *BRCA1/2* mutations, a total of 37 and 29 *BRCA1/2* mutation variants were identified from OV and BROV cohorts, respectively. The number of Asian‐specific BRCA mutations identified in the BROV cohort (13.8%, 4/29) were more than that in the OV cohort (5.4%, 2/37) (Figure S1). In both cohorts, around 8%–10% of novel mutations were identified (Table S4). The list of pathogenic variants in both cohorts were shown in Table S4.

### 3.3 Disease‐free survival and overall survival of OV cohort

Cox proportional hazard model of DFS and OS analysis of *BRCA1/2* mutation group were conducted on OV cohort with adjustment of diagnosis age, stage, and cancer grade. There was no significant difference between the *BRCA1/2* mutation group and the negative group (DFS *p* value = .7440, OS *p* value = .8044) while stage and grade showed a significant effect on both survivals (Tables 2 and 3). DFS and OS analysis on the BROV cohort was not feasible, owing to the small sample size and the event definition cannot be well defined. Our preliminary data did not confine to observations in larger cohorts. A study of 3979 patients with invasive epithelial ovarian cancer, from there, 1232 having a germline mutation in *BRCA1/2*, showed that patients having mutations in *BRCA1/2* was associated with an improved 5‐year overall survival, and there was a better prognosis for *BRCA2* carriers (Bolton et al., 2012). In our small sample (*N* = 45), our analysis is vulnerable to confounding variables that affect prognosis, such as age, cancer staging and/or grading, specific histology of ovarian cancer or chemotherapy regime, etc. Statistical techniques such as stratified survival analyses might not be particularly useful in this small cohort.

**TABLE 2:** Cox proportional hazard model on DFS on OV cohort

Variable	Odds ratio	*p* value
Mutation (reference = Negative)		
*BRCA* mutation positive	1.0823	.7440^a^
Stage (reference = 1)		
2	2.4209	.0089
3	6.4571	<.0001
Grade (reference = 1)		
2	1.3719	.5151
3	2.6410	.0237
Diagnosis age	1.0108	.2626

^a^ Mutation is not statistically significant when taking other variables into account.

**TABLE 3:** Cox proportional hazard model on OS on OV cohort

Variable	Odds ratio	*p* value
Mutation (reference = Negative)		
*BRCA* mutation positive	1.1174	.8044^a^
Stage (reference = 1)		
2	7.9501	.0179
3	15.0848	.0004
4	27.4904	.0001
Grade (reference = 1)		
2	2.4433	.4249
3	4.0505	.1824
Diagnosis age	0.9654	.0508

^a^ Mutation is not statistically significant when taking other variables into account.

## 4 DISCUSSION

This study estimated the prevalence of gene mutations in patients with OV and patients with both BROV. Overall, 12.8% of OV cancer patients carried a mutation and a higher prevalence was seen in BROV cancer patients (40.9%). A high proportion of mutations were in *BRCA1/2* genes, they were identified in 10% of OV cancer patients and 35.5% of BROV cancer patients. A high *BRCA1/2* mutation rate of 38.9% has also been demonstrated in Taiwan in a cohort of 18 synchronous/metachronous BROV cancer studies (Chao et al., 2020). *BRCA1/2* mutations (14.6%–22.8%) were more likely found in high‐grade serous carcinoma of ovarian than other types of ovarian cancer (Alsop et al., 2012; Candido‐dos‐Reis et al., 2015; Harter et al., 2016). We have a slightly higher *BRCA* mutation rate, 28.8% of our ovarian cancer cases diagnosed with high grade serous were *BRCA1/2* positive carcinoma when compared with those in other Asian countries, such as study on 207 epithelial ovarian cancers from Japan (Sugino et al., 2019), and a study on 313 epithelial ovarian cancers from Korea (Seo et al., 2019), both showed 20% and 20.9% *BRCA1/2* positive in high‐grade serous carcinoma. It is, however, noted that our patients were from a younger cohort (aged 9–78) than those in Japan (aged 32–89) and Korea (aged 42–58). This may be due to the differences in the health care system and cultural variations in these regions, younger patients are allowed to seek medical attention in our locality, hence, identification of much younger patients.

*BRCA1/2* mutations were the most common mutation identified in this study. There was no specific genomic regional clustering for the mutations in *BRCA1*. However, mutations in *BRCA2* were mostly located at c.3109C > T (Kwong et al., 2012). Among 37 types of *BRCA1/2* mutation variants identified in the OV cohort, 34 of them have previously been identified in our local breast cancer cohort (Kwong et al., 2016). This group of patients had no significant difference between the *BRCA1/2* mutation group and the mutation‐negative group in DFS and OS with adjustment of diagnosis age, stage, and cancer grade. Note that our data conflicts with the literature including a study conducted by Bolton et al. (2012). Their paper reported that among patients with invasive epithelial ovarian cancer, having a germline mutation in *BRCA1/2* was associated with improved 5‐year overall survival and that *BRCA2* showed the best prognosis. A possible explanation may be of small sample size; confounding variables that affect prognoses such as age, cancer stage or grade, histology of ovarian cancer or chemotherapy regime, etc. The differences in clinical features of *BRCA1/2* mutation carriers and those patients with mutations in genes other than *BRCA1/2* remained to be established in larger cohorts. It is, however, clear that detection of *BRCA1/2* mutation is the most important gene to be screened in these patients. In centers with limited resources, *BRCA1/2* and a small panel of related genes might be considered for preliminary screening.

In genes other than the *BRCA1/2* panel, *MSH2* is a mismatch repair gene known to be conferring susceptibility to ovarian cancer in Lynch Syndrome. The lifetime risk for ovarian cancer in families with Lynch syndrome is ~8% (Nakamura et al., 2014). *MSH2* was the most common mutated gene other than *BRCA1/2* in both the OV cohort (4.6%) and the BROV cohort (4.7%). Patients in both cohorts had similar mutation rates in *MSH2*. In a study of 279 women with proven Lynch syndrome, 10% of the ovarian patients carried a *MSH2* mutation (Ryan et al., 2017). OV was identified in 14% of these *MSH2* mutation carriers and most cases were diagnosed before age 50 (Møller et al., 2017).

The second commonly mutated genes were *PALB2* in the OV cohort (1.8%) and the BROV cohort (4.7%). It is noted that there was no *MSH2* and *PALB2* mutation identified in a 207 ovarian cancer study among Japanese (Sugino et al., 2019). To date, there is no large‐scale study has been performed to compare the mutation frequencies in patients with OV and those with BROV. The high mutation rate of *PALB2* and *MSH2* in the BROV cohort denoted the importance of extended panel sequencing. Individual comparison on the characteristics of non‐BRCA genes mutation carriers was not feasible due to limited numbers of positive carriers in the two cohorts.

Interestingly, mutations in *RAD51* genes, *RAD51C* (0.9%) and *RAD51D* (2.8%) were only seen in the OV cohort but not in the BROV cohort. In another study evaluating the contribution of *RAD51* genes, small proportions of pathogenic mutations in *RAD51C* (14/3429, 0.41%) and *RAD51D* (12/3429, 0.35%) in invasive epithelial ovarian cancer were identified (Song et al., 2015). In another study, 1.5% (3/207) *RAD51* mutations were identified in Japanese ovarian cancer patients (Sugino et al., 2019). Our previous study showed that low frequencies of *RAD51C* (0.08%) and *RAD51D* mutations (0.73%) were identified in cohorts of breast cancers only (Kwong et al., 2020). These suggested that *RAD51* gene mutations are associated with a higher risk of ovarian cancers than breast cancers.

In future development and consideration on cost‐effectiveness, patients with only ovarian cancer worth using the extended panel as the primary screening while for patients with BROV cancers, stepwise strategy with a primary screening on the small panel then extended panel exemplified a cost‐effective business model in clinical NGS sequencing for hereditary cancer conditions. The main aim would still be trying to increase the size of the screening net so as to increase the number of patients and family members for counseling and further surgical surveillance and management. In this study, we have only shown the prognostic contributions of *BRCA1/2* mutations in OV cancers patients and patients with both BROV cancers. The contributions of mutations in genes other than *BRCA*s need to be established in future studies as we expanded our screening strategy.

In conclusion, our data provide insight into the germline mutation spectrum of patients with OV only, and those with both BROV cancers. The study confirms a high prevalence of *BRCA1/2* mutations in both cohorts. The strategy involving initial screening of common 4 genes, followed by an extended screening of the negative cases was cost‐effective in patients with BROV cancers but not in patients with ovarian cancers only. In a situation where cost or limited resources is concerned, ovarian cancer patients with serous epithelial ovarian carcinoma or patients with both personal BROV cancers should be selected for screening. The spectrum of mutations identified is important in genetic counseling.

## CONFLICT OF INTEREST

The authors declare that they have no competing interests.

## AUTHOR CONTRIBUTIONS

The study was designed by AK, VS, TC, KC, HN, and EM. CH performed the experiments, collected data, and drafted the manuscript. CA performed the bioinformatics analysis. WL and LF assisted in statistical analysis. AK, VS, TC, KC, HN, and EM reviewed the manuscript. All authors read and approved the final manuscript.

## ETHICS APPROVAL AND PATIENT CONSENT

The study was performed according to the Declaration of Helsinki. Written informed consent was obtained from participants recruited in this study. This study was approved by the Institutional Review Board of the University of Hong Kong/Hospital Authority West Cluster and respective authorities of other contributing hospitals in Hong Kong. All methods were performed in accordance with the relevant guidelines and regulations.

## Supporting information


Figure S1

Table S1

## Data Availability

Data available in article supplementary material.
