# Supplementary material for: Germline mutations in Chinese ovarian cancer with or without breast cancer
Source: Mol Genet Genomic Med. 2022 May 24;10(7):e1940. doi: 10.1002/mgg3.1940 (PMC9266594; doi:10.1002/mgg3.1940)
Supplement: Supplementary file 1 — Figure S1 [file MGG3-10-e1940-s001.docx]

**Supplementary Figure 1. *BRCA*s mutation variants seen in the ovarian cancer (OV) cohort and breast and ovarian cancer (BROV) cohort.**

Common; Novel; Asia specific.
