# Supplementary material for: Germline mutations in Chinese ovarian cancer with or without breast cancer
Source: Mol Genet Genomic Med. 2022 May 24;10(7):e1940. doi: 10.1002/mgg3.1940 (PMC9266594; doi:10.1002/mgg3.1940)
Supplement: Supplementary file 2 — Table S1 [file MGG3-10-e1940-s002.docx]

**Supplementary Table 1a. Clinicopathologic characteristics of ovarian cancer patient’s (OV) cohort.**

| **OV patients** | **Total (n=451)** | **Non mutation carries (n=392)** | **Mutation carriers (n=59)** | **p-value**  **(pos vs neg)** |
| --- | --- | --- | --- | --- |
| **Age, Median**  **(range, yr)** | 47 (9-78) | 47.5 (9-78) | 45 (17-74) | 0.7404 |
| **Diagnosis age <50** | 261 (57.8%) | 227 (57.9%) | 34 (57.6%) | 1 |
| **Ovarian Cancer Stage** | | | | |
| I | 192 (44%) | 186 (49.2%) | 6 (10.3%) | <0.0001 |
| II | 58 (13.3%) | 52 (13.8%) | 6 (10.3%) |  |
| III | 139 (31.9%) | 109 (28.8%) | 30 (51.7%) |  |
| IV | 47 (10.8%) | 31 (8.2%) | 16 (27.6%) |  |
| Not stated | 15 | 14 | 1 |  |
| **Ovarian Cancer Site** | | | | |
| Ovarian | 407 (90.2%) | 359 (91.6%) | 48 (81.4%) | 0.0456 |
| Fallopian tube | 9 (2%) | 6 (1.5%) | 3 (5.1%) |  |
| Peritoneal | 27 (6%) | 21 (5.4%) | 6 (10.2%) |  |
| Synchronous cancer | 8 (1.8%) | 6 (1.5%) | 2 (3.4%) |  |
| **Ovarian Cancer Histology** | | | | |
| Epithelial | 421 (96.3%) | 364 (95.8%) | 57 (100%) | 1 |
| Germ cell | 5 (1.1%) | 5 (1.3%) | 0 (0%) |  |
| Stromal | 5 (1.1%) | 5 (1.3%) | 0 (0%) |  |
| Synchronous cancer | 6 (1.4%) | 6 (1.6%) | 0 (0%) |  |
| Not stated | 14 | 12 | 2 |  |
| **Ovarian Cancer Histology Subtype** | | | | |
| Serous | 161 (36.9%) | 118 (31.1%) | 43 (75.4%) | <0.0001 |
| Non serous | 275 (63.1%) | 261 (68.9%) | 14 (24.6%) |  |
| Not stated | 15 | 13 | 2 |  |
| **Ovarian Cancer Histology Subtype** | | | | |
| Endometrioid | 145 (33.3%) | 137 (36.1%) | 8 (14%) | 0.0008 |
| Non Endometrioid | 291 (66.7%) | 242 (63.9%) | 49 (86%) |  |
| Not stated | 15 | 13 | 2 |  |
| **Ovarian Cancer Grade** | | | | |
| 1 | 48 (11.1%) | 47 (12.5%) | 1 (1.8%) | <0.0001 |
| 2 | 111 (25.6%) | 108 (28.6%) | 3 (5.4%) |  |
| 3 | 274 (63.3%) | 222 (58.9%) | 52 (92.9%) |  |
| Not stated | 18 | 15 | 3 |  |
| **Family History of Cancers** | | | | |
| Breast | 92 (20.4%) | 71 (18.1%) | 21 (35.6%) | 0.0031 |
| Ovarian | 27 (6.0%) | 17 (4.3%) | 10 (16.9%) | 0.0010 |
| BRCA related | 140 (31%) | 113 (28.8%) | 27 (45.8%) | 0.0105 |

**Supplementary Table 1b. Clinicopathologic characteristics of breast and ovarian cancer patient’s (BROV) cohort.**

| **BR&OV patients** | **Total (n=93)** | **Non mutation carries (n=55)** | | **Mutation carriers**  **(n=38)^§^** | **p-value**  **(pos vs neg)** |
| --- | --- | --- | --- | --- | --- |
| **Age, median**  **(range, yr)** | 45 (18-73) | 48 (18-64) | | 43 (22-73) | 0.0299 |
| **Diagnosis age <50** | 62 (66.7%) | 30 (54.5%) | | 32 (84.2%) | 0.0035 |
| **Ovarian Cancer Stage** | | | | | |
| Stage I | 37 (48.1%) | 26 (63.4%) | | 11 (30.6%) | 0.0209 |
| Stage II | 4 (5.2%) | 2 (4.9%) | | 2 (5.6%) |  |
| Stage III | 28 (36.4%) | 11 (26.8%) | | 17 (47.2%) |  |
| Stage IV | 8 (10.4%) | 2 (4.9%) | | 6 (16.7%) |  |
| Not stated | 16 | 14 | | 2 |  |
| **Ovarian Cancer Site** | | | | | |
| Ovarian | 79 (84.9%) | 50 (90.9%) | | 29 (76.3%) | 0.1267 |
| Fallopian tube | 2 (2.2%) | 1 (1.8%) | | 1 (2.6%) |  |
| Peritoneal | 6 (6.5%) | 1 (1.8%) | | 5 (13.2%) |  |
| Uterus | 6 (6.5%) | 3 (5.5%) | | 3 (7.9%) |  |
| **Ovarian Cancer Histology** | | | | | |
| Epithelial | 84 (96.6%) | 47 (94%) | | 37 (100%) | 0.5055 |
| Germ cell | 2 (2.3%) | 2 (4%) | | 0 (0%) |  |
| Stromal | 1 (1.1%) | 1 (2%) | | 0 (0%) |  |
| Not stated | 6 | 5 | | 1 |  |
| **Ovarian Cancer Histology Subtype** | | | | | |
| Serous | 42 (48.8%) | 14 (28.6%) | | 28 (75.7%) | <0.0001 |
| Non serous | 44 (51.2%) | 35 (71.4%) | | 9 (24.3%) |  |
| Not stated | 7 | 6 | | 1 |  |
| **Ovarian Cancer Histology Subtype** | | | | | |
| Endometrioid | 24 (27.9%) | 16 (32.7%) | | 8 (21.6%) | 0.3338 |
| Non Endometrioid | 62 (72.1%) | 33 (67.3%) | | 29 (78.4%) |  |
| Not stated | 7 | 6 | | 1 |  |
| **Ovarian Cancer Grade** | | | | | |
| 1 | 9 (11.3%) | 7 (15.6%) | | 2 (5.7%) | 0.0435 |
| 2 | 11 (13.8%) | 9 (20%) | | 2 (5.7%) |  |
| 3 | 60 (75%) | 29 (64.4%) | | 31 (88.6%) |  |
| Not stated | 13 | 10 | | 3 |  |
| **Breast Cancer Stage** | | | | | |
| Stage 0 | 16 (18.2%) | 13 (24.1%) | 3 (8.8%) | | 0.135 |
| Stage I | 30 (34.1%) | 14 (25.9%) | 16 (47.1%) | |  |
| Stage II | 30 (34.1%) | 20 (37%) | 10 (29.4%) | |  |
| Stage III | 11 (12.5%) | 6 (11.1%) | 5 (14.7%) | |  |
| Stage IV | 1 (1.1%) | 1 (1.9%) | 0 (0%) | |  |
| Not stated | 5 | 1 | 4 | |  |
| **Breast Cancer Histology** | | | | | |
| Ductal | 60 (70.6%) | 31 (60.8%) | 29 (85.3%) | | 0.0649 |
| Non-ductal | 9 (10.6%) | 7 (13.7%) | 2 (5.9%) | |  |
| In situ | 16 (18.8%) | 13 (25.5%) | 3 (8.8%) | |  |
| Not stated | 8 | 4 | 4 | |  |
| **Breast Cancer Grade** | | | | | |
| Low | 44 (58.7%) | 31 (67.4%) | 13 (44.8%) | | 0.0599 |
| High | 31 (41.3%) | 15 (32.6%) | 16 (55.2%) | |  |
| Not stated | 18 | 9 | 9 | |  |
| **Breast Cancer Molecular Subtype** | | | | | |
| HER2+ | 7 (7.5%) | 5 (9.1%) | 2 (5.3%) | | 0.6963 |
| ER/PR+ | 40 (43.0%) | 29 (52.7%) | 11 (28.9%) | | 0.0328 |
| TNBC | 15 (16.1%) | 3 (5.5%) | 12 (31.6%) | | 0.0012 |
| **Family History of Cancer** | | | | | |
| Breast | 36 (38.7%) | 15 (27.3%) | 21 (55.3%) | | 0.0092 |
| Ovarian | 14 (15.1%) | 4 (7.3%) | 10 (26.3%) | | 0.0173 |
| BRCA related | 29 (31.2%) | 12 (21.8%) | 17 (44.7%) | | 0.0239 |

**^§^**Two probands carried double mutation

**Supplementary Table 2a. Comparing the mutation frequency of *BRCA*s and genes other than *BRCAs* in ovarian (OV) cohort.**

|  | **Ovarian cancer probands (N=451)** | | | **p-value** | |
| --- | --- | --- | --- | --- | --- |
|  | **BRCA mutation carriers**  **(n=45)** | **Beyond BRCA mutation carriers**  **(n=14)** | **Non mutation carries (n=392)** | **BRCA mutation**  **VS**  **Negative** | **Beyond BRCA mutation VS Negative** |
| **Age, median (range, yr)** | 50 (17-74) | 42.5 (30-59) | 47.5 (9-78) | 0.4413 | 0.0316 |
| **Diagnosis age < 50** | 22 (48.9%) | 12 (85.7%) | 227 (57.9%) | 0.2682 | 0.0508 |
| **Ovarian Cancer Stage** | | | | | |
| Stage I | 2 (4.5%) | 4 (28.6%) | 186 (49.2%) | <0.0001 | 0.0633 |
| Stage II | 4 (9.1%) | 2 (14.3%) | 52 (13.8%) |  |  |
| Stage III | 26 (59.1%) | 4 (28.6%) | 109 (28.8%) |  |  |
| Stage IV | 12 (27.3%) | 4 (28.6%) | 31 (8.2%) |  |  |
| Not stated | 1 | 0 | 14 |  |  |
| **Ovarian Cancer Site** | | | | | |
| Ovary | 35 (77.8%) | 13 (92.9%) | 359 (91.6%) | 0.0130 | 0.3913 |
| Fallopian tube | 3 (6.7%) | 0 (0%) | 6 (1.5%) |  |  |
| Peritoneal | 6 (13.3%) | 0 (0%) | 21 (5.4%) |  |  |
| Mixed | 1 (2.2%) | 1 (7.1%) | 6 (1.5%) |  |  |
| **Ovarian Cancer Histology** | | | | | |
| Epithelial | 45 (100%) | 12 (100%) | 364 (95.8%) | 1 | 1 |
| Germ cell | 0 (0%) | 0 (0%) | 5 (1.3%) |  |  |
| Stromal | 0 (0%) | 0 (0%) | 5 (1.3%) |  |  |
| Mixed | 0 (0%) | 0 (0%) | 6 (1.6%) |  |  |
| Not stated | 0 | 2 | 12 |  |  |
| **Ovarian Cancer Histology Subtype** | | | | | |
| Serous | 39 (86.7%) | 4 (33.3%) | 118 (31.1%) | <0.0001 | 1 |
| Non Serous | 6 (13.3%) | 8 (66.7%) | 261 (68.9%) |  |  |
| Not stated | 0 | 2 | 13 |  |  |
| **Ovarian Cancer Histology Subtype** | | | | | |
| Endometrioid | 3 (6.7%) | 5 (41.7%) | 137 (36.1%) | <0.0001 | 0.7637 |
| Non Endometrioid | 42 (93.3%) | 7 (58.3%) | 242 (63.9%) |  |  |
| Not stated | 0 | 2 | 13 |  |  |
| **Ovarian Cancer Grade** | | | | | |
| 1 | 0 (0%) | 1 (8.3%) | 47 (12.5%) | <0.0001 | 0.6952 |
| 2 | 1 (2.3%) | 2 (16.7%) | 108 (28.6%) |  |  |
| 3 | 43 (97.7%) | 9 (75%) | 222 (58.9%) |  |  |
| Not stated/0 | 1 | 2 | 15 |  |  |
| **Family History of Cancer** | | | | | |
| Breast | 17 (37.8%) | 4 (28.6%) | 71 (18.1%) | 0.005 | 0.3024 |
| Ovarian | 9 (20.0%) | 1 (7.1%) | 17 (4.3%) | 0.0005 | 0.4756 |
| BRCA related | 16 (35.6%) | 11 (78.6%) | 113 (28.8%) | 0.3887 | 0.0002 |

**Supplementary Table 2b. Comparing the mutation frequency of *BRCA*s and genes other than *BRCAs* in breast and ovarian (BROV) cohort.**

|  | **Breast and ovarian cancer probands (N=93¶)** | | | **p-value*** | |
| --- | --- | --- | --- | --- | --- |
|  | ***BRCA* mutation carriers (n=33)** | **Beyond *BRCA* mutation carriers (n=6)** | **Non mutation carries (n=55)** | ***BRCA* mutation VS Negative** | **Beyond *BRCA* mutation VS Negative** |
| **Age, median (range, yr)** | 43 (30-73) | 38 (22-46) | 48 (18-64) | 0.0941 | 0.0943 |
| **Diagnosis age < 50** | 27 (81.8%) | 6 (100.0%) | 30 (54.5%) | 0.0244 | 0.0692 |
| **Ovarian Cancer Stage** | | | | | |
| Stage I | 7 (21.2%) | 4 (66.7%) | 26 (63.4%) | 0.0038 | 1 |
| Stage II | 2 (6.1%) | 0 (0%) | 2 (4.9%) |  |  |
| Stage III | 16 (48.5%) | 2 (33.3%) | 11 (26.8%) |  |  |
| Stage IV | 6 (18.2%) | 0 (0%) | 2 (4.9%) |  |  |
| Not stated | 2 | 0 | 14 |  |  |
| **Ovarian Cancer Site** | | | | | |
| Ovary | 27 (81.8%) | 3 (50%) | 50 (90.9%) | 0.0339 | 0.0183 |
| Fallopian tube | 1 (3%) | 0 (0%) | 1 (1.8%) |  |  |
| Peritoneal | 5 (15.2%) | 0 (0%) | 1 (1.8%) |  |  |
| Uterus | 0 (0%) | 3 (50%) | 3 (5.5%) |  |  |
| **Ovarian Cancer Histology** | | | | | |
| Epithelial | 32 (100%) | 6 (100%) | 47 (94%) | 0.7033 | 1 |
| Germ cell | 0 (0%) | 0 (0%) | 2 (4%) |  |  |
| Stromal | 0 (0%) | 0 (0%) | 1 (2%) |  |  |
| Not stated | 1 | 0 | 5 |  |  |
| **Ovarian Cancer Histology Subtype** | | | | | |
| Serous | 27 (84.4%) | 2 (33.3%) | 14 (28.6%) | <0.0001 | 1 |
| Non Serous | 5 (15.6%) | 4 (66.7%) | 35 (71.4%) |  |  |
| Not stated | 1 | 0 | 6 |  |  |
| **Ovarian Cancer Histology Subtype** | | | | | |
| Endometrioid | 4 (12.5%) | 4 (66.7%) | 16 (32.7%) | 0.0640 | 0.0570 |
| Non Endometrioid | 28 (87.5%) | 2 (33.3%) | 33 (67.3%) |  |  |
| Not stated | 1 | 0 | 6 |  |  |
| **Ovarian Cancer Grade** | | | | | |
| 1 | 1 (3.3%) | 1 (16.7%) | 7 (15.6%) | 0.0020 | 0.4145 |
| 2 | 0 (0%) | 2 (33.3%) | 9 (20%) |  |  |
| 3 | 29 (96.7%) | 3 (50%) | 29 (64.4%) |  |  |
| Not stated/0 | 3 | 0 | 10 |  |  |
| **Family History of Cancer** | | | | | |
| Breast | 21 (63.6%) | 1 (16.7%) | 15 (27.3%) | 0.0016 | 0.3180 |
| Ovarian | 10 (30.3%) | 1 (16.7%) | 4 (7.3%) | 0.0126 | 1 |
| BRCA related | 14 (42.4%) | 3 (50.0%) | 12 (21.8%) | 0.0508 | 0.0943 |
| **Breast Cancer Stage** | | | | | |
| Stage 0 | 1 (3.4%) | 2 (33.3%) | 13 (24.1%) | 0.0380 | 0.7299 |
| Stage I | 14 (48.3%) | 2 (33.3%) | 14 (25.9%) |  |  |
| Stage II | 9 (31%) | 2 (33.3%) | 20 (37%) |  |  |
| Stage III | 5 (17.2%) | 0 (0%) | 6 (11.1%) |  |  |
| Stage IV | 0 (0%) | 0 (0%) | 1 (1.9%) |  |  |
| Not stated | 4 | 0 | 1 |  |  |
| **Breast Cancer Histology** | | | | | |
| Ductal | 26 (89.7%) | 4 (66.7%) | 31 (60.8%) | 0.0163 | 0.8179 |
| Non-ductal | 2 (6.9%) | 0 (0%) | 7 (13.7%) |  |  |
| In situ | 1 (3.4%) | 2 (33.3%) | 13 (25.5%) |  |  |
| Not stated | 4 | 0 | 4 |  |  |
| **Breast Cancer Grade** | | | | | |
| Low | 11 (44%) | 2 (40%) | 31 (67.4%) | 0.1225 | 0.5973 |
| High | 14 (56%) | 3 (60%) | 15 (32.6%) |  |  |
| Not stated | 8 | 1 | 9 |  |  |
| **Breast Cancer Molecular Subtype** | | | | | |
| HER2+ | 2 (6.1%) | 0 (0%) | 5 (9.1%) | 1 | 1 |
| ER/PR+ | 8 (24.2%) | 3 (50.0%) | 29 (52.7%) | 0.014 | 1 |
| TNBC | 12 (36.4%) | 0 (0%) | 3 (5.5%) | 0.0002 | 1 |

**¶**One of the patient carried both BRCA and PALB2 mutation, clinical data was counted in both BRCA and Beyond BRCA group but was excluded in Fisher’s exact test.

**Supplementary Table 3a. *BRCA*s mutation rate by age in cancer groups**

|  | | ***BRCA*s+_OV** | | ***BRCA*s+_BR**^‡^ | | ***BRCA*s+_BR&OV** | | **p-value** |
| --- | --- | --- | --- | --- | --- | --- | --- | --- |
|  | | **N** | **%** | **N** | **%** | **N** | **%** |  |
| **Age of Dx** | **0-19** | 1/9 | 11.11 | 0/3 | 0.00 | 0/1 | 0.00 | 1 |
|  | **20-44** | 15/158 | 9.49 | 136/1462 | 9.30 | 20/44 | 45.45 | <0.0001 |
|  | **45-64** | 26/257 | 10.12 | 78/967 | 8.07 | 12/47 | 25.53 | 0.0010 |
|  | **>=65** | 3/27 | 11.11 | 4/187 | 2.14 | 1/1 | 100.00 | 0.0026 |

^‡^Data from Hong Kong Heredity Breast Cancer Family Registry

**Supplementary Table 3b. *BRCA* mutations in different cancers carrying families.**

| **Cancer Family** | ***BRCA* mutation carriers** | ***BRCA***  **Negative** | **p-value** | **Beyond *BRCA* mutation carriers** | **Beyond *BRCA* tested negative** | **p-value** |
| --- | --- | --- | --- | --- | --- | --- |
| Ovarian (OV) | 28 (8.0%) | 321 (92.0%) | p<0.001 | 10 (11.9%) | 74 (88.1%) | p=0.001 |
| Breast^§^ | 176 (7.3%) | 2238 (92.7%) |  | 45 (3.9%) | 1095 (96.1%) |  |
| Breast & Ovarian (BROV)^∥^ | 91 (29.7%) | 215 (70.3%) |  | 10 (8.6%) | 106 (91.4%) |  |

^§^Data from Kwong A *et al*., 2020 and Hong Kong Heredity Breast Cancer Family Registry

^∥^One of the patient from BROV cohort carried both BRCA and PALB2 mutation was excluded in the above calculation.
